# MoMo30 Binds to SARS-CoV-2 Spike Variants and Blocks Infection by SARS-CoV-2 Pseudovirus

**DOI:** 10.3390/v16091433

**Published:** 2024-09-07

**Authors:** Kenya DeBarros, Mahfuz Khan, Morgan Coleman, Vincent C. Bond, Virginia Floyd, Erick Gbodossou, Amad Diop, Lauren R. H. Krumpe, Barry R. O’Keefe, Michael D. Powell

**Affiliations:** 1 Department of Microbiology, Biochemistry and Immunology, Morehouse School of Medicine, 720 Westview Dr. SW, Atlanta, GA 30310, USA; kdebarros@msm.edu (K.D.); mkhan@msm.edu (M.K.); mcoleman@msm.edu (M.C.); vbond@msm.edu (V.C.B.); 2 Department of Community Health and Preventive Medicine, 720 Westview Dr. SW, Atlanta, GA 30310, USA; vfloyd@msm.edu; 3 PROMETRA International, Dakar-Etoile BP 6134, Senegal; erick.gbodossou@gmail.com; 4 Malango Traditional Healers Association, Fatick BP 1763, Senegal; mikepowell5072@gmail.com; 5 Molecular Targets Program, Center for Cancer Research, National Cancer Institute, Frederick, MD 21702-1201, USA; lauren.haughkrumpe@nih.gov (L.R.H.K.); okeefeba@mail.nih.gov (B.R.O.); 6 Natural Products Branch, Developmental Therapeutic Program, Division of Cancer Treatment and Diagnosis, National Cancer Institute, Frederick, MD 21702, USA

**Keywords:** COVID-19, SARS-CoV-2, Pseudovirus, MoMo30, fusion inhibitor, extract, antiviral, antiviral protein, antiviral plant, traditional medicine, ethnopharmacology

## Abstract

MoMo30 is an antiviral protein isolated from aqueous extracts of *Momordica balsamina* L. (Senegalese bitter melon). Previously, we demonstrated MoMo30’s antiviral activity against HIV-1. Here, we explore whether MoMo30 has antiviral activity against the COVID-19 virus, SARS-CoV-2. MLV particles pseudotyped with the SARS-CoV-2 Spike glycoprotein and a Luciferase reporter gene (SARS2-PsV) were developed from a three-way co-transfection of HEK293-T17 cells. MoMo30’s inhibition of SARS2-PsV infection was measured using a luciferase assay and its cytotoxicity using an XTT assay. Additionally, MoMo30’s interactions with the variants and domains of Spike were determined by ELISA. We show that MoMo30 inhibits SARS2-PsV infection. We also report evidence of the direct interaction of MoMo30 and SARS-CoV-2 Spike from WH-1, Alpha, Delta, and Omicron variants. Furthermore, MoMo30 interacts with both the S1 and S2 domains of Spike but not the receptor binding domain (RBD), suggesting that MoMo30 inhibits SARS-CoV-2 infection by inhibiting fusion of the virus and the host cell via interactions with Spike.

## 1. Introduction

The study of traditional medicines and their uses in various cultural heritages is known as ethnopharmacology [1]. Traditional medicine should not be conflated with alternative medicine. As Fontanarosa, P. B., and Lundberg, G. D.,1998 [2] explain, “There is no alternative medicine. There is only scientifically proven, evidence-based medicine supported by solid data or unproven medicine for which scientific evidence is lacking. Whether a therapeutic practice is ‘Eastern’ or ‘Western,’ is unconventional or mainstream, or involves mind-body techniques or molecular genetics is largely irrelevant except for historical purposes and cultural interest.” One group of plants used in traditional medicine is the genus Momordica.

Momordica, also known as bitter melons, is a genus of plants from the family, Cucurbitaceae [3]. Most of the scientific literature on bitter melons pertains to *Momordica charantia.* Bitter melons are commonly used as traditional remedies in countries such as Brazil, China, Ghana, Haiti, India, Mexico, and New Zealand [4]. *M. charantia* is often studied extensively as an herbal medicine for type 2 diabetes [5,6]. In Togo, the general population and traditional healers report a high degree of *M. charantia*’s use in treating gastrointestinal and viral diseases [5]. Traditional uses of bitter melons also include measles, hepatitis, wounds, fevers, and gastrointestinal and viral diseases [4,7]. Phytochemicals isolated from *M. charantia*, such as alpha and beta momorachrins, have also been studied for their anti-HIV activity [5,8,9]. The attention put on the medicinal uses of *M. charantia* has overshadowed the scientific evaluation of almost all other bitter melons, including the Senegalese bitter melon *M. balsamina*.

In Senegal, traditional health practitioners brew a tea of the *M. balsamina* leaves and administer it orally to treat infectious diseases [10,11]. Because of this and other notable examples of Senegalese ethnopharmacology, a deliberate effort is being made to preserve and scientifically evaluate the conventional applications of their traditional medicine. One such organization engaged in this effort is our collaborator, the PROmotion of MEdicine and TReatment from Africa (PROMETRA International), which is based in Senegal and facilitates the relationship between modern and traditional medicine. PROMETRA promotes treatments that have been shown to be safe and effective while discouraging those that are not [10,12].

We have previously shown the anti-HIV properties of aqueous extracts of *M. balsamina* [10,11]. Our lab isolated extracts of *M. balsamina* through methods described in Coleman et al., 2022 [10] and identified the active agent as a protein approximately 30 kDa in weight that we have named MoMo30. Considering that MoMo30 comes from a bitter melon, is an approximately 30 kDa protein, and has anti-HIV activity, we considered that MoMo30 might be the previously reported protein, MAP30, which is a ribosome inactivating protein (RIP) that comes from *M. charantia* and *M. balsamina*. However, MAP30 is thought to target later steps of the viral replication cycle by inactivating ribosomes and/or the HIV viral genome, preventing their replication, transcription, and translation. [7,13]. By contrast, MoMo30 targets viral fusion, the earliest step of the cycle. Furthermore, a BLAST alignment of the MoMo30 and MAP30 sequence revealed that there was no significant sequence similarity between them. The closest sequence match to MoMo30 is actually most closely related to Hevamine A from the White Carob tree, *Prosopis alba*, with a 93% sequence identity [11]. Therefore, MoMo30 is a previously unknown, Hevamine A-like protein.

In Khan et al., 2023 [11], we determined that MoMo30 inhibits HIV infection by binding to gp120, the surface glycoprotein of HIV, to prevent the fusion of HIV to its host cell. With the recent Coronavirus Infectious Disease-2019 (COVID-19) pandemic, we sought to determine if MoMo30 could also bind to the glycoprotein of the Severe Acute Respiratory Syndrome Coronavirus 2 (SARS-CoV-2) and inhibit infection by acting on the Spike protein in a fashion similar to HIV.

COVID-19 is caused by the coronavirus SARS-CoV-2. Most infections remain mild, with up to 20–40% of patients being asymptomatic. Mild symptoms of COVID-19 include fever, cough, and loss of smell and taste. However, some patients experience more severe disease caused by a “cytokine storm” that causes acute respiratory distress syndrome (ARDS) and potentially life-threatening cell death to host tissues [14]. According to the World Health Organization (WHO), there have been over 775 million confirmed cases of COVID-19, including over 7 million deaths as of May 2024 [15].

Coronaviruses are enveloped viruses containing a non-segmented, positive-sense, single-stranded RNA [16]. All coronaviridae possess a surface envelope glycoprotein, Spike, which binds to the host cell receptor, angiotensin converting enzyme 2 (ACE2), to initiate viral fusion [17]. Therefore, Spike is an identified target of current vaccines against SARS-CoV-2 and a potential target for antiviral drug development. 

The initial antiviral strategy against COVID-19 was the repurposing of other drugs such as lopinavir, nelfinavir, and ritonavir (initially designed for HIV), Arbidol (designed for influenza), and ribavirin (commonly used for Hepatitis C) [18]. Anti-parasitic drugs such as ivermectin and hydroxychloroquine were also tested as potential COVID treatments in April 2020 [19,20] but proved ineffective. Despite the failure of ivermectin and hydroxychloroquine, misinformation and misreporting in the media on these two drugs led to their “panic purchasing” by consumers before a scientific consensus was reached with regard to their effectiveness. Hydroxychloroquine was later shown to be ineffective at treating COVID-19 [21] and the data regarding ivermectin were inconclusive [22]. For this reason, neither of these two drugs gained FDA approval for treating COVID-19.

In October 2020, the FDA did, however, approve the use of Veklury (remdesivir), which was initially developed for Hepatitis C, as a treatment for COVID-19 [23,24]. By December 2020, the Morbidity and Mortality Weekly Report (MMWR) announced the FDA approval of COVID vaccines developed by Pfizer and Moderna [25,26]. As of December 2021, two antivirals received FDA emergency use authorization for COVID-19, the protease inhibitor, Paxlovid (nirmatrelvir and ritonavir) [27], and RNA polymerase inhibitor, Lagevrio (molnupiravir) [28]. There has yet to be an approved antiviral drug that targets the SARS-CoV-2 Spike protein. The search for an antiviral drug that targets the SARS-CoV-2 Spike could benefit from the scientific evaluation of traditional medicines. In the current study, we demonstrate the antiviral activity of the protein MoMo30 against a SARS-CoV-2 and its interactions with the SARS2 Spike protein from several SARS-CoV-2 variants, and hypothesize on the potential mechanism of action of this antiviral protein.

## 2. Materials and Methods

### 2.1. Production of Crude M. balsamina Extract

Methods for processing plant materials and creating a raw extract of MoMo30 were first outlined in Coleman et al., 2022 [10]. Briefly, M. balsamina was grown and harvested in Dakar-Etoile, Senegal, by our partners at PROMETRA International. Dried leaves were crushed using a plant mill then added to water. The mixture was boiled at 100 °C for 30 min and air-dried into a powder before shipping. A tea was made from 100 g of M. balsamina powder dissolved in 1 L of sterile distilled water at 4 °C overnight. It was then extensively filtered using a Whatman Grade 3 filter paper (Whatman Cat# 1003-240, Whatman, Maidstone, England) and centrifuged at 4000× *g* for 30 min to remove potential particulate contamination. We sterilized the extract using a 0.45 micron filter and then lyophilized it into a powder for storage at −20 °C.

### 2.2. Ammonium Sulfate Protein Precipitation

MoMo30 was purified initially using an ammonium sulfate precipitation method as described in O’Keefe et al., 1997 [29]. Briefly, the lyophilized crude aqueous extract was brought up to 25 mg/mL in water and placed on ice. Dry, crystalline ammonium sulfate (Sigma–Aldric Cat# A915-5KG, Sigma Aldrich, Darmstadt, Germany) was added to the sample and brought up to a 75–80% saturation. The samples were incubated on ice for 1 h and then precipitated overnight at 4 °C. The samples were centrifuged at 3000 rpm for 30 min. The supernatant was decanted and discarded, and the remaining pellet was reconstituted in 1 mL of DD H2O and passed through a 10 kDa Vivaspin ultrafiltration filter (GE Healthcare Cat# 28-9323-60) and washed 3 times with up to 1 mL of water to remove any remaining ammonium sulfate. 

### 2.3. Isolation of MoMo30 through Fast Protein Liquid Chromatography (FPLC)

Following precipitation, the protein was brought up to 1 mL in the starting buffer and injected onto a 1 mL HiTrap Capto Phenyl ImpRes Column (Cytiva, Cat# 17548411, Marlborough, MA, USA) previously equilibrated with a starting buffer of 50 mM sodium phosphate, 1.5 M ammonium sulphate, pH 7.0. FPLC was performed on a Bio-Rad NGC chromatography system. Following application of the sample, the column was washed in a 6 mL starting buffer, followed by a 40 mL gradient elution of the proteins using the starting buffer and the elution buffer, 50 mM sodium phosphate, pH 7.0. The proteinaceous column eluates were pooled together, desalted, and concentrated using a 10 kDa Vivaspin Filter (GE Healthcare, Cat# 28-9323-60, Atlanta, GA, USA), filtering low molecular weight proteins and retaining MoMo30. The purity of the MoMo30 sample was verified with SDS-PAGE.

### 2.4. SARS2—Pseudovirus Production

The basis for the Pseudovirus (PsV) assay system used to determine activity against SARS-CoV-2 is outlined in Xu et al., 2022 [30]. Briefly, SARS2 PsV were produced via transfection of HEK293T/17 cells (ATCC, Cat# CRL-11268) which were maintained in Dulbecco-modified eagle medium (DMEM) with L-Glutamine and phenol red (Gibco, Cat# 11965-092, New York, NY, USA) supplemented with (1%) penicillin–streptomycin (1% Gibco, Cat# 15140-122) and 10% Fetal Bovine Serum (FBS) (Hyclone Cat# SH30070.03, Logan, UT, USA) at 37 °C under 5% CO_2_. HEK-293T/17 cells were cultured 5 × 10^6^ cells per tissue culture flask and incubated overnight to allow cell attachment to the flaks. After overnight attachment, the medium was replaced with 6 mL, pre-warmed OptiMEM (Gibco, Cat# 11058-021, New York, NY, USA) per flask for 30 min prior to transfection.

Transfection was carried out according to the Lipofectamine 3000 manufacturer’s protocol (Thermo Fisher Cat# L3000001, Waltham, MA, USA). A three-way co-transfection of the cells was carried out with the following plasmids: 6 μgpackaging plasmid MLV gag/pol:pTG 5349 (TransGene via NCATS, Illkerch-Graffenstaden, France), 8 μg transfer vector encoding luciferase, MLV Ψ-RNA signal, and MLV LTR: pTG13077 (NCATS), and 6 μg SARS-CoV-2 S glycoprotein: NR-52310 (BEI Resources, Manassas, VA, USA). Flasks were incubated for 4 h (37 °C) followed by a gentle addition of 7 mL complete medium (without antibiotics or phenol red). After a 2-day incubation, SARS-CoV-2 PsV were harvested from the flask media, passed through a 0.45 μm cellulose acetate filter (Sartorius S6555-FMSUK, Göttingen, Germany), aliquoted, and stored at −80 °C.

### 2.5. SARS2-Pseudovirus Assay

The SARS2-PsV assay was carried out using HEK293T/ACE2 (HEK293T cells expressing human ACE2-BEI Resources Cat# NR-52511, Gaithersburg, MD, USA) maintained in Dulbecco-modified eagle medium (DMEM) with L-glutamine and phenol red (Gibco, Cat# 11965-092, NewYork, NY, USA) supplemented with (1%) penicillin–streptomycin (Gibco, Cat# 15140-122) and 10% fetal bovine serum (FBS) (Hyclone Cat# SH30070.03, Logan, UT, USA) at 37 °C under 5% CO_2_.

HEK293/ACE2 cells were seeded onto a 96-well black tissue culture plate, 1000 cells/well in 68 µL of medium containing DMEM (Gibco, Cat# 31053-028), 10% FBS (Hyclone, Cat# SH30070-03, Logan, UT, USA), 1% l-glutamine (Gibco, Cat# 25030-081), and 1% Pen–strep, (Gibco, Cat# 15140-122, New York, NY, USA). Following an overnight incubation, 16 µL of 10× polybrene, 16 µL of MoMo30 at different doses or control compounds, followed by 60 µL of the SARS-CoV-2 PsV prep were added sequentially. The plates were briefly spun to adhere SARS-CoV-2 PsV to the cell monolayer and incubated for 1 h. Following a 3-day incubation, the plates were centrifuged at 1000× *g* for 20 min. For the luciferase-infectivity assay, 100 µL of the Neolite luciferase reagent (Perkin–Elmer Cat# 6016716, Waltham, MA, USA) was added to each well and read for luminescence. 

### 2.6. XTT Cytotoxicity Assay

HEK293/ACE2 cells were seeded onto a 96-well clear tissue culture plate, infected, and treated in tandem with PsV assay to measure cytotoxicity arising from test samples. A 1 mg/mL of sodium 3′-[1-(phenylaminocarbonyl)-3,4-tetrazolium]-bis (4-methoxy6-nitro) benzene sulfonic acid hydrate (XTT) assay solution was prepared from XTT powder (NSC Cat# 601519, Frederick, MD, USA) and warm medium without phenol red. Phenazine methosulfate (PMS) (Sigma–Aldrich Cat# P9625, St. Louis, MO, USA) was added 4% to the XTT solution. Immediately, 50 µL/well of the final solution was added to the plate, followed by a 4-h incubation at 37 °C. The plates were read for absorbance at 450 nm. Samples that have a color, like crude extracts of MoMo30, were found to alter the color of the media and interfere with readings in luminescent and colorimetric assays. Because of this, the media of each well was carefully replaced with 100 µL of fresh, pre-warmed media before adding the luciferase reagent and XTT solution.

### 2.7. Spike Variant and Domain ELISAs

Methods for performing the enzyme-linked immunosorbent assays (ELISAs) are outlined in Munoz-Basagoiti et al., 2023 [31]. Briefly, purified, recombinant SARS-CoV-2 Spike protein WH-1 (BPS Bioscience Cat #100728, San Diego, CA, USA), Alpha, Delta, and Omicron Spike proteins (Protein Expression Laboratory, FNLCR, Frederick, MD, USA) were each produced in HEK293 cells and immobilized on high-binding ELISA plates. For evaluating the binding of MoMo30 to Spike variants, plates were washed three times with phosphate buffered saline with 0.01% Tween-20 (PBS-T) and incubated with serial half-log dilutions of MoMo30, diluted in PBS. For evaluating the binding of MoMo30 to the domains of the Spike glycoprotein, the S1 domain (Protein Expression Laboratory, FNLCR, Frederick, MD, USA), the S2 domain (Millipore Sigma, Cat AGX820, Burlington, MA, USA), and the receptor binding domain (RBD, a sub-domain within the S1 domain) (R&D Biosystems, 10500-cv-100, Minneapolis, MN, USA) were used according to the same protocol. For both the variant and domain ELISAs, the negative control (the absence of any kind of Spike) was subtracted as background from the figures.

## 3. Results

### 3.1. Crude Extracts of MoMo30 Can Inhibit a SARS2 Pseudovirus

**Figure 1:**
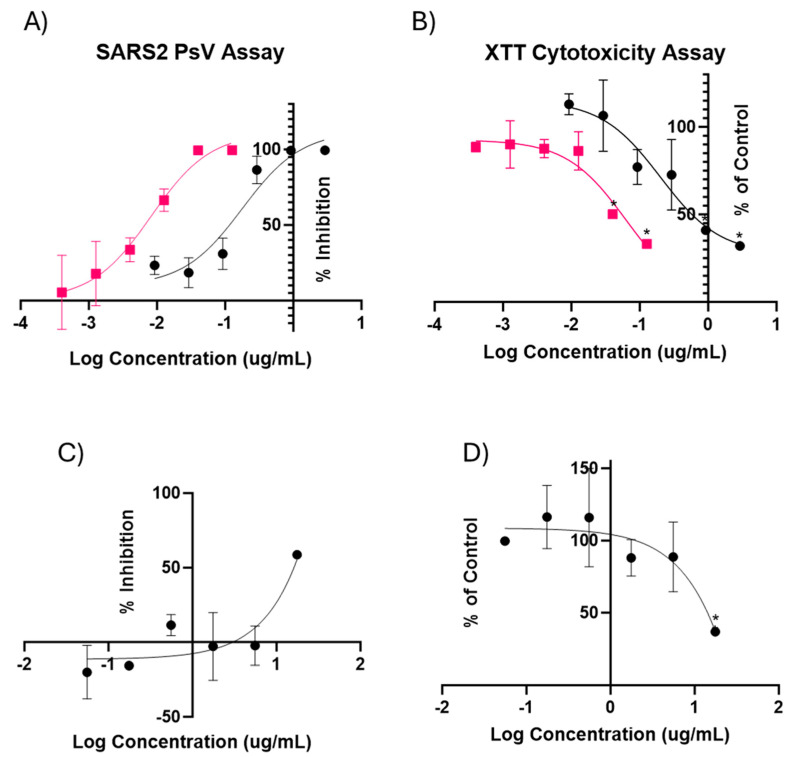
(**A**) SARS2-PsV assay. This assay was conducted in triplicate, generating a dose–response curve measuring the viral inhibition of the crude (●) and ammonium sulfate precipitated extracts (■) of MoMo30 against SARS2-PsV. (**B**) XTT Cytotoxicity assay. The assay was conducted in tandem with the PsV assay with the percent cell viability compared with non-treated controls for each concentration shown. The (*) indicate statistically significant difference (*p* < 0.05) in cytotoxicity between treated on non-treated controls. (**C**) SARS2-PsV assay of *M. balsamina* tannins. This is a dose–response curve measuring the viral inhibition of the isolated tannins against SARS2-PsV. Treatment concentrations range from 0 to 17.61 μg/mL. (**D**) XTT Cytotoxicity assay of *M. balsamina* tannins. Concentrations of tannins are in the same dose range. The (*) indicate statistically significant difference (*p* < 0.05) in cytotoxicity between treated on non-treated controls. The assays were conducted in triplicate. The mean and SD are shown.

To determine if water-soluble extracts of *M. balsamina* contained potential anti-SARS-CoV-2 activity, we infected HEK293-ACE2 cells with SARS-CoV-2 PsV and treated them with varying concentrations of crude extracts of MoMo30 and semi-pure extracts produced from ammonium sulfate precipitation. Treatment concentrations ranged from 0 to 2.91 μg/mL (IC50 = 0.179 μg/mL) and 0 to 0.127 μg/mL (IC50 = 8.22 ng/mL) of crude and precipitated extracts, respectively. MoMo30 was mixed with virus and directly added to cells and incubated at 37 °C for 72 h. The MoMo30 extracts inhibited PsV infection at all doses. The results are summarized in Figure 1A. The data were analyzed by non-linear regression of the infectivity results using GraphPad Prism version 10.0.2 for Windows, GraphPad Software, Boston, MA, USA, www.graphpad.com accessed on 1 May 2024.

### 3.2. XTT Cytotoxicity of Crude and Precipitated Extracts of MoMo30

Cytotoxicity of *M. balsamina* leaf extract was determined using the XTT Assay. The crude extract was not toxic at inhibitory levels up to 0.291 μg/mL. Cells treated with up to 0.012 μg/mL of precipitated extract also showed anti-viral activity and were non-toxic. However, cytotoxicity increased significantly in the cells treated with 0.925 μg/mL of crude extract and 0.04 μg/mL of precipitated extract. We show the results for the XTT assay in Figure 1B using the same non-linear regression analysis method as used for antiviral activity data. Cytotoxic doses were determined using a Brown Forsythe and Welch ANOVA that compares the cytotoxicity of treated cells with non-treated controls.

### 3.3. SARS2 Pseudovirus and XTT Assay of M. balsamina Tannins

It should be noted that the tannins present in the crude and precipitated samples prevent all proteins, even those <10 kDa, from passing through the spin filter. Tannins have been shown to have some antiviral activity on their own [32,33]. Therefore, it stood to reason that the antiviral activity of the *M. balsamina* extract could be attributed to its tannins. The extract’s tannins that were isolated from FPLC were also tested in the infectivity and cytotoxicity assays (Figure 1C,D). The tannins have little to no antiviral activity on their own with a dose of 17.6 μg/mL being cytotoxic. This eliminates tannins as the source of the extract’s antiviral activity.

### 3.4. MoMo30’s Interactions with Spike Variants

**Figure 2:**
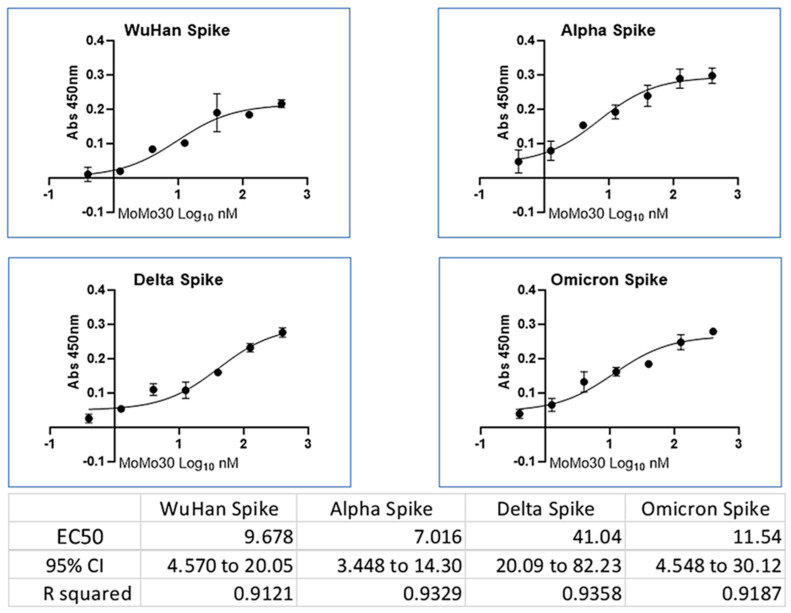
Spike Variant ELISA. MoMo30 binds to the SARS-CoV-2 Spike glycoprotein Wu Han-1, Alpha, Delta, and Omicron BA.1 Spike variants. The assays were performed in triplicate. The mean and SD are shown.

After the demonstration of anti-viral activity of MoMo30 against SARS-CoV-2 PsV, multiple ELISA experiments were conducted to examine the binding of MoMo30 to the Spike glycoprotein of the original Wu Han (WH-1) strain of SARS-CoV-2. The same experiment sought to detect the binding of MoMo30 to the Spike proteins belonging to the Alpha, Delta, and Omicron BA.1 variants of SARS-CoV-2. The results indicated that MoMo30 binds the Spike of all four variants with a slight preference for the Alpha Spike glycoprotein (see Figure 2).

### 3.5. MoMo30’s Interactions with Spike Domains

**Figure 3:**
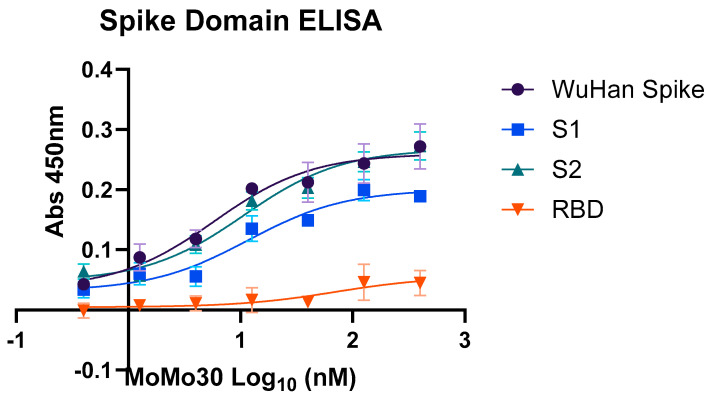
MoMo30 binds to full-length SARS-CoV-2 spike glycoprotein, the isolated S1 and S2 domains of Wu Han Spike, but not to the isolated receptor binding domain (RBD). The assays were done in triplicate. The mean and SD are shown.

To further elucidate the MoMo30–Spike interactions, we also tested the binding of MoMo30 to different domains of Spike. The SARS-CoV-2 Spike, unlike that of SARS-CoV-1, contains a Furin-cleavage site which delineates the S1 (AAs 1 to 614) and S2 (AAs 615 to 1273) domains of the Spike glycoprotein [17]. The S1 domain contains the receptor binding domain (RBD) which binds ACE2 on human cells. After being cleaved, the S2 domain facilitates the fusion of the viral envelope with the host cell’s membrane, thus infecting the cell [17]. We therefore evaluated the binding of MoMo30 to the RBD, S1, and S2 domains of Spike by ELISA. We show that MoMo30 binds to full-length9 Spike proteins and the isolated S1 and S2 domains but, interestingly, not the RBD (Figure 3).

## 4. Discussion

It has been shown that MoMo30 inhibits the entry of HIV through specific binding to oligomannoside structures decorating the surface of gp120 [11]. Additionally, when Rhesus macaques were orally administered *M. balsamina* extracts, MoMo30 was found in the serum of the macaques after 42 days of administration, indicating that MoMo30 is orally bioavailable [11]. This serves as a model for antiviral activity in HIV and a potential model for COVID-19 patients experiencing viremia. For patients that do not develop viremia, MoMo30 might not reduce SARS-CoV-2 entry unless the compound is available at the corresponding sites of replication within the respiratory tract. Although MoMo30’s availability in the respiratory tract is unknown, we speculate that since MoMo30 is so stable, it might be possible to formulate it for inhalation in order to target replication sites within the respiratory tract.

With the COVID-19 pandemic in 2020, we endeavored to assess the potential use of MoMo30 against SARS-CoV-2 infections seeing that, like HIV, it is an enveloped virus with a surface glycoprotein bearing mannosylated glycans. Similarly to HIV, MoMo30 prevents viral entry by interactions with SARS-CoV-2 by binding to the viral surface glycoprotein, suggesting that MoMo30 functions as an entry inhibitor. The fact that MoMo30 did not bind to the isolated RBD domain suggests that MoMo30 does not inhibit initial viral attachment but instead inhibits a later aspect of viral entry. Due to its oral bioavailability and ability to address an, as yet unexploited, target (the Spike protein), MoMo30 is a potential therapeutic agent to treat COVID-19. The results of our ELISA data demonstrate that MoMo30’s possible mechanism of action is related to its interactions with the domains of Spike. This leads us to three hypotheses for MoMo30’s potential mechanism of action. 

**Figure 4:**
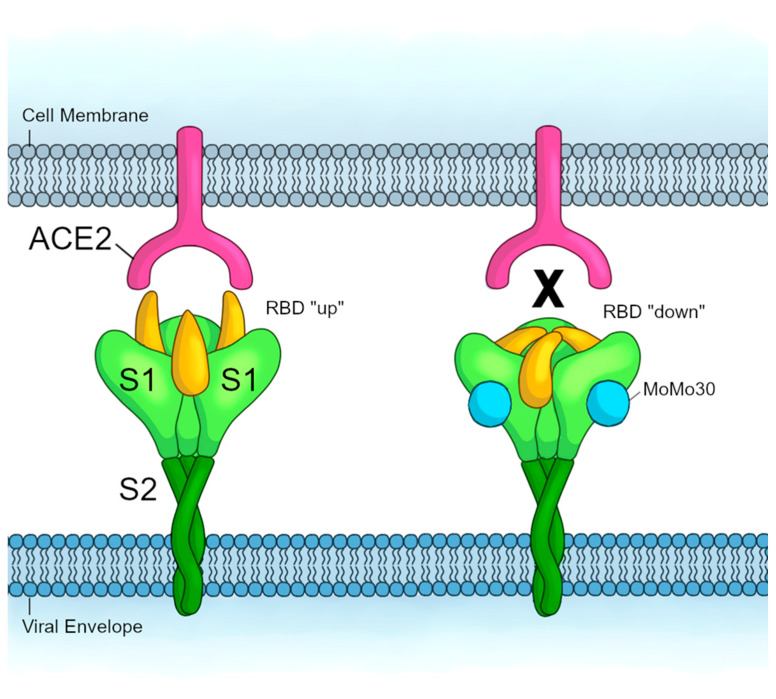
S1 Conformation Inhibition Hypothesis. The Spike protein consists of the S1 (light green) and S2 (dark green) domains. The RBD (yellow) adopts an “up” conformation when binding to the ACE2 receptor (pink) to allow viral attachment to the host cell. MoMo30 (blue) binds to the S1 domain such that the RBD is stuck in the “down” conformation and cannot attach to ACE2.

The first hypothesis is the “S1 conformation inhibition hypothesis.” In our ELISA of the isolated domains of Spike, MoMo30 binds S1 but showed very little binding to the RBD within S1. However, it has been established that the RBD of S1 is flexible and adopts an “up” conformation when binding to ACE2. The carboxyterminal domain 1 (CTD1), fusion-peptide proximal region (FPPR), amino-terminal domain (NTD), and the 630 loop are subdomains within S1 that have to shift to accommodate the RBD’s movement into the “up” conformation [34]. Although MoMo30 binds to both the S1 and S2 domains, in this hypothesis, MoMo30’s true antiviral activity lies within its interactions with the subdomains of S1 such that it sterically inhibits S1 domain conformational flexibility. The RBD is stuck in the “down” conformation and cannot bind ACE2 for fusion to occur. In this case, MoMo30 would prevent viral attachment to the host cell (See Figure 4). 

**Figure 5:**
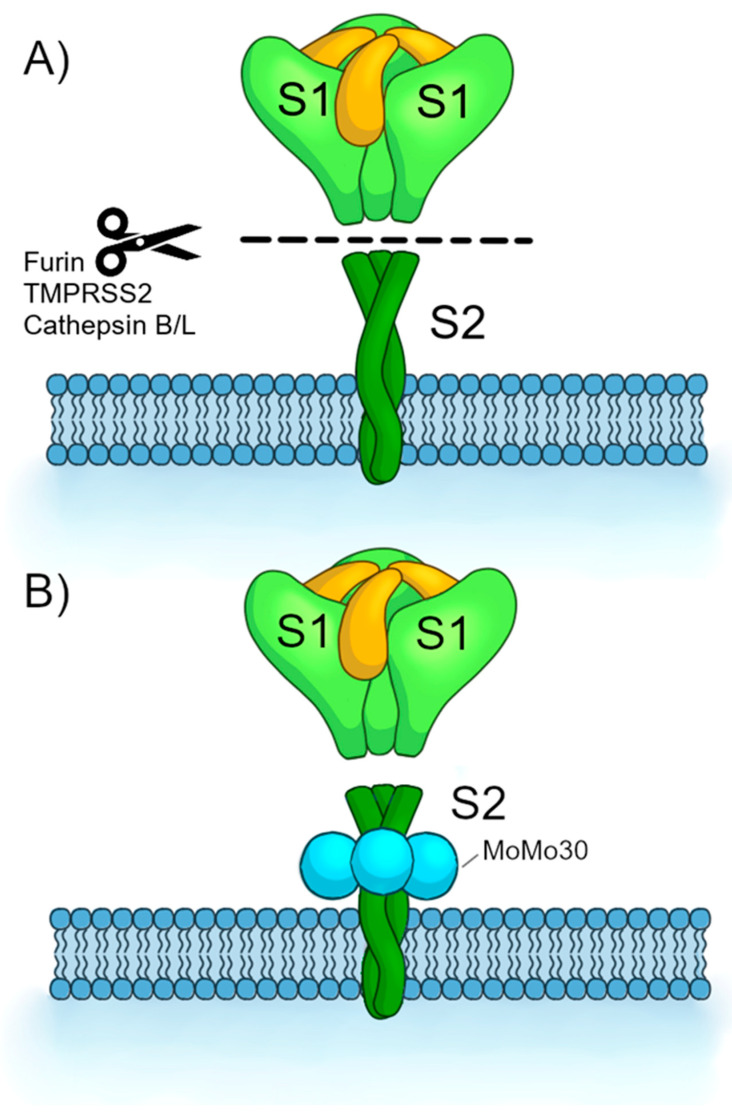
S2 Conformation Inhibition Hypothesis. (**A**) Spike protein is cleaved by host cell proteases (represented with a pair of scissors) and releases the S1 domain. (**B**) MoMo30 binds the S2 domain and inhibits the necessary conformation changes in the S2 for fusion to occur.

The second hypothesis is the “S2 conformation inhibition hypothesis.” It has also been established that once the S1 domain is released from the S2, a domain within the S2 called the “fusion peptide” is exposed. The fusion peptide physically inserts into the host cell membrane. Then, S2 undergoes a conformation change driven by its heptad repeat and central helix domains such that the viral envelope and cell membrane are joined together and thus fusion occurs [35]. In this hypothesis, MoMo30’s antiviral activity lies with its interactions within the S2 domain such that it sterically inhibits the S2 domain. Therefore, MoMo30 prevents the S2 from performing the conformation change necessary for fusion (See Figure 5). In this case, MoMo30 would not prevent viral attachment but still prevent viral entry.

**Figure 6:**
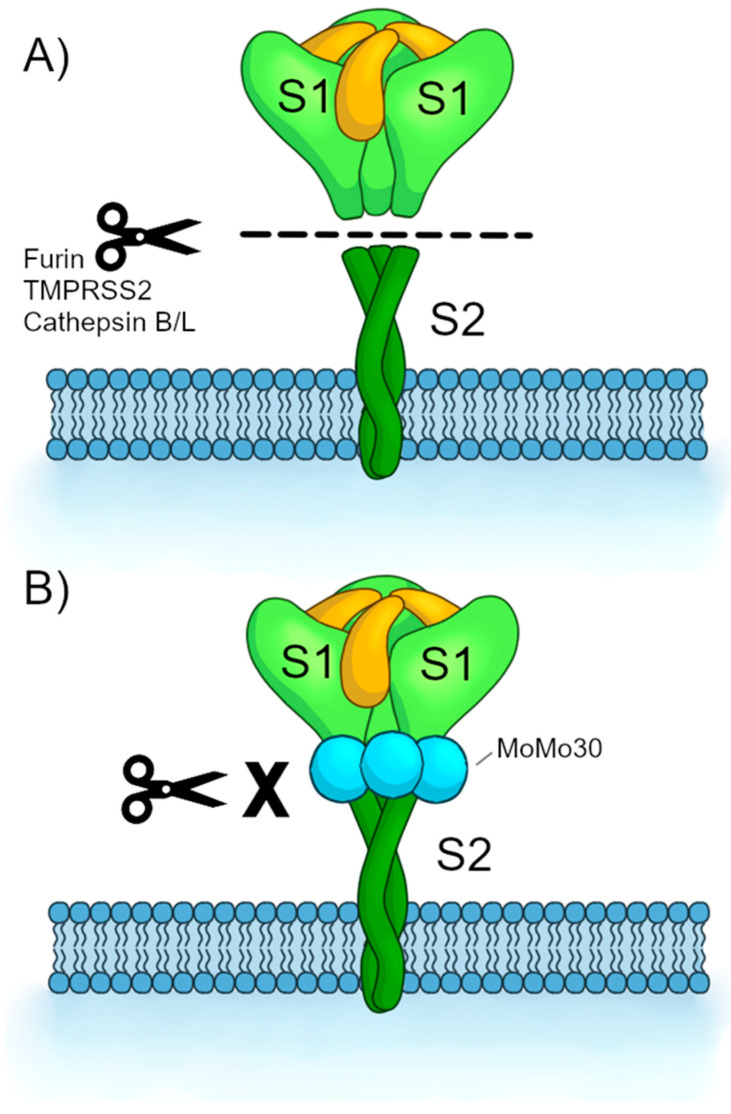
Protease Inhibition Hypothesis. (**A**) Spike protein is cleaved by host cell proteases along the boundary between the S1 and S2 domains. The release of the S1 domain exposes the fusion peptide within the S2. (**B**) MoMo30 blocks the cleavage by the proteases.

The third is the “Protease Inhibition Hypothesis.” It has been established that viral fusion is a multistep process with the proteolytic cleavage of SARS-CoV-2 Spike by host cell proteases as an essential step after the binding of Spike to ACE-2 [17]. Host cell proteases such as Furin, TMPRSS2, and Cathepsin B/L can release the S1 domain from the S2 by proteolytic cleavage at the S1/S2 or S2’ boundary [35]. Given that MoMo30 binds the S1 and S2 domains (as shown by ELISA), it is possible that MoMo30 binds at or in the vicinity of these cleavage sites, thus preventing the necessary cleavage of Spike (See Figure 6). Furthermore, there are reported high mannose bearing oligosaccharides at positions on or near Spike’s cleavage sites between amino acids 603 and 801 [36]. Because MoMo30 binds the high mannose oligosaccharides of gp120 [11], it would stand to reason that MoMo30 binds the Spike cleavage sites via the same mechanism.

Although Furin, TMPRSS2, and Cathepsin B/L all cleave Spike, they do so at different stages in the viral replication cycle. Furin, which is localized in the Trans-Golgi network, cleaves Spike at the S1/S2 boundary as newly made virions are assembled and exit the cell. This ensures that some of the virus’s Spikes are pre-primed for viral fusion upon infection of a new cell. TMPRSS2, localized to the plasma membrane, also cleaves Spike but at the S2’ site to expose the fusion peptide. Alternatively, if the target cell expresses little TMPRSS2, the virus can be internalized via clathrin-mediated endocytosis into the endolysosomes, where S2′ cleavage is performed by cathepsins [34]. However, the virus relies on direct cell surface entry more than endosomal entry; therefore, we anticipate that TMPRSS2 is the protease that is most likely to be inhibited by MoMo30 in this hypothesis.

The proteolytic cleavage and “fusion” conformation of a viral surface protein is not unique to the SARS-CoV-2 Spike. The surface proteins of other enveloped viruses such as, but not limited to, HIV, Influenza, Herpes Simplex Virus, and Ebola also function this way [37], each with their own cleavage recognition sequences (see Table 1). Given that MoMo30 has inhibitory effects against both the SARS-2 and HIV through interactions with Spike and gp120, respectively, it is possible that MoMo30 could also employ the same mechanism of action across multiple other viruses, suggesting that MoMo30 could be a broad-spectrum antiviral in the future or at least pave the way for drugs that also target via this mechanism. Antiviral research often focuses on the receptor binding domains of viral surface proteins. However, the work achieved here with MoMo30 demonstrates that the cleavage sites and “hinges” of these proteins should also be of interest in rational design.

In all three of these hypotheses, MoMo30 binds to Spike via its oligosaccharides. As of yet, there is no direct experimental evidence to show that MoMo30 and Spike bind in this manner; however, we anticipate that they do based on our experiments that show MoMo30 and gp120 binding in this manner. Verifying MoMo30’s interactions with Spike’s glycans would require the testing of MoMo30’s ability to bind a deglycosylated Spike protein. These hypotheses could be tested through solving the crystal structure of MoMo30, which also remains unknown. However, we anticipate that the co-crystallization of MoMo30 and Spike would likely be difficult. In our experience, lectins typically have multi-valent interactions with viral glycoproteins which causes them to aggregate and produce protein crystals with a poor mosaicity. Performing cryo-electron microscopy on these protein aggregates would be a challenge for the same reason. It would be more beneficial to co-crystallize MoMo30 with an oligosaccharide and study their interactions that way. Furthermore, MoMo30’s ability to inhibit the enzymatic activity of Furin, TMPRSS2, and Cathepsin B/L in cleaving full-length Spike would have to be tested.

## 5. Patents

This work is covered by the US patents: US20220054601A1, US11925670B2, US 11,266,723 B1, and US 11,414,462.

## Figures and Tables

**Table 1:** Cleavage Sites of Viral Glycoproteins. Despite being different viruses with different glycoproteins and receptors, they share the common trait of relying on proteolytic cleavage for infection to occur. Obtained from [35,37].

Virus	Glycoprotein	Protease	Sequence Recognition Site
SARS-CoV-1	Spike	TMPRSS2 Cathepsin B/L	_796_KR
SARS-CoV-2	Spike	Furin TMPRSS2	_732_RRAR _864_KR
HIV-1	Env Gp120	PCSK7 Furin	_508_REKR
Ebola	GP sGP	Furin	_497_RRTRR _321_RVRR
Influenza (H7N1)	HA	Furin TMPRSS2 TMPRSS4	_337_KKREKR

## Data Availability

The data in this study are not publicly available due to patent considerations but are available on request.
